# Photon and electron backscatter dose and energy spectrum analysis around Lipiodol using flattened and unflattened beams

**DOI:** 10.1002/acm2.12560

**Published:** 2019-03-18

**Authors:** Daisuke Kawahara, Shuichi Ozawa, Tomoki Kimura, Akito Saito, Takeo Nakashima, Yoshimi Ohno, Yuji Murakami, Yasushi Nagata, Takehiro Shiinoki

**Affiliations:** ^1^ Radiation Therapy Section Department of Clinical Support Hiroshima University Hospital Hiroshima Japan; ^2^ Department of Radiation Oncology Institute of Biomedical & Health Sciences Hiroshima University Hiroshima Japan; ^3^ Department of Radiation Oncology Graduate School of Medicine Yamaguchi University Yamaguchi Japan

**Keywords:** backscatter, dose enhancement, Lipiodol

## Abstract

**Purpose:**

The aim of the current study was to evaluate the backscatter dose and energy spectrum from the Lipiodol with flattening filter (FF) and flattening filter‐free (FFF) beams. Moreover, the backscatter range, that was defined as the backscatter distance (BD) are revealed.

**Methods:**

6 MVX FF and FFF beams were delivered by TrueBeam. Two dose calculation methods with Monte Carlo calculation were used with a virtual phantom in which the Lipiodol (3 × 3 × 3 cm^3^) was located at a depth of 5.0 cm in a water‐equivalent phantom (20 × 20 × 20 cm^3^). The first dose calculation was an analysis of the dose and energy spectrum with the complete scattering of photons and electrons, and the other was a specified dose analysis only with scattering from a specified region. The specified dose analysis was divided into a scattering of primary photons and a scattering of electrons.

**Results:**

The lower‐energy photons contributed to the backscatter, while the high‐energy photons contributed the difference of the backscatter dose between the FF and FFF beams. Although the difference in the dose from the scattered electrons between the FF and FFF beams was within 1%, the difference of the dose from the scattered photons between the FF and FFF beams was 5.4% at a depth of 4.98 cm.

**Conclusions:**

The backscatter range from the Lipiodol was within 3 mm and depended on the Compton scatter from the primary photons. The backscatter dose from the Lipiodol can be useful in clinical applications in cases where the backscatter region is located within a tumor.

## 1 INTRODUCTION

Several institutions have recently reported promising responses from patients with unresectable hepatocellular carcinoma treated with trans arterial chemoembolization (TACE) and followed by stereotactic body radiation therapy (SBRT).^1^, ^2^ Lipiodol in which each milliliter contained 480 mg of iodine organically combined with the ethyl esters of fatty acids of poppy seed oil has remained in SBRT treatment and it is useful for image‐guided radiation therapy (IGRT) and dose enhancement in the tumor region. Dawson et al. proposed that tumors pretreated by TACE with Lipiodol can be used for direct targeting in IGRT.^3^ In our previous paper, we reported the dose enhancement and energy spectrum in Lipiodol with FF and FFF beams.^4^, ^5^ We revealed the factor of the dose enhancement that was defined as the probability of electron generation. However, this study did not evaluate the backscatter dose from the Lipiodol. The backscatter around high‐Z materials, that is dental implants, results in local dose enhancement. The backscatter dose enhancement affects the normal tissue dose. Its prevention has clinical interest and has been investigated extensively.^6^, ^7^, ^8^ Çatli reported that a high atomic number and density of dental implants led to major problems with regard to providing an accurate dose distribution in radiotherapy, and also with regard to contouring tumors caused by artifacts in head and neck tumors.^9^ They showed that the large errors were caused for the treatment planning without Monte Carlo calculation (MC). The MC method is a good approach toward deriving the dose distribution in heterogeneous media. The Lipiodol is also high‐Z material, thus the backscatter dose and range from the Lipiodol should be evaluated for the tumor and normal tissue such as the normal liver, duodenum, and intestine. Moreover, Chin reported that dental restorations, fixed prosthodontics, and implants affect the dose distribution in head and neck radiation therapy owing to the high atomic number of the materials used.^10^ The researchers concluded that backscatter from high‐Z materials affects the occurrence of mucositis in the treatment of head and neck cancers. Although they investigated local dose enhancement with various high‐Z materials, a detailed analysis of the backscatter dose with regard to the effect of primary photons and scattered electrons has not yet been carried out.

Medical linear accelerators capable of generating flattening filter‐free (FFF) beams have recently been developed. The FFF beam increases the dose delivery efficiency of state‐of‐the‐art radiotherapy techniques.^11^ The removal of the flattening filter largely decreases the attenuation of the beam. Cashmore reported that the FFF beam contains low‐energy photons, which contribute to the dose deposition in the photon beam buildup region close to the surface of the patient's body.^12^ The results of our previous study revealed that these lower photons contributed to dose enhancement in the Lipiodol region for the FFF beam.^5^

The purpose of current study was to analyze the backscatter dose and energy spectrum from the Lipiodol with FF and FFF beams. Furthermore, we reveal the correlation of the backscatter dose and distance by analyzing the specified dose from the Lipiodol.

## 2 MATERIALS AND METHODS

A Varian TrueBeam linac (Varian Medical Systems, Palo Alto, USA) with 6‐MV x‐ray (6X) FF and FFF beams was used. The components of the TrueBeam accelerator head are proprietary and not commercially available for direct simulations. However, the phase space wherein Varian provides the IAEA‐compliant phase‐space files that were simulated using the GEANT4 MC code was scored onto the surface of a cylinder located above the secondary collimator. The phase‐space files below the secondary collimator were modeled with BEAMnrc code.^13^ Phase‐space data scored at a source‐to‐surface distance (SSD) of 90 cm were used as the input data for an inhomogeneous virtual phantom. Dose calculations and photon and electron energy spectra acquisitions were performed by MC code named the Particle and Heavy Ion Transport code System (PHITS).^14^ Moreover, PHITS had a specified dose calculation function that could only calculate the scattered dose from the specified region. The dose calculation grid size behind the buildup region was 0.5 × 0.5 × 0.5 mm^3^. The cutoff energies for the photons and electrons were set to 0.01 and 0.7 MeV, respectively. The number of photon histories in BEAMnrc and PHITS was 2.0 × 10 ^8^ and 4.0 × 10 ^9^, respectively. The verification of the MC calculation was performed with the data measured by a CC04 (IBA Dosimetry, TN, USA) chamber of volume 0.04 cm^3^.

**Figure 1:**
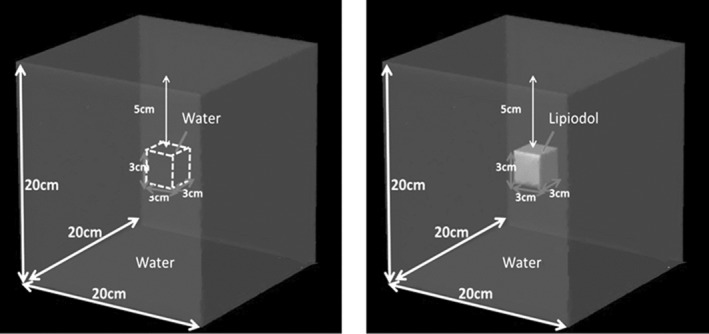
Geometric scheme of Lipiodol located at depth of 5.0 cm in water‐equivalent phantom.

The dose difference around the Lipiodol, between the doses in the phantoms with and without Lipiodol, was evaluated by a simple virtual phantom. A virtual inhomogeneous phantom in which Lipiodol (3 × 3 × 3 cm^3^) was located at a depth of 5.0 cm in a water‐equivalent phantom (20 × 20 × 20 cm^3^) was made (Fig. 1). The mass density of Lipiodol was overridden by 1.28 g/cm^3^. A field size of 5 × 5 cm^2^ was used to irradiate at SSD = 90 cm. The depth dose was calculated and normalized to the calculated dose at D_max_. The energy spectral variations of the photons and electrons were investigated by the same beam and virtual phantom that was used in the analysis of the dose difference. The number of bins in each spectrum was set to 50, with the energy ranging from 0 to 20 MeV. The energy spectrum was analyzed at a depth of 4.98 cm, immediately behind the Lipiodol, with and without the Lipiodol. Additionally, the energy spectrum was normalized to the dose per monitor unit (MU) with 6X FF and FFF beams.

**Figure 2:**
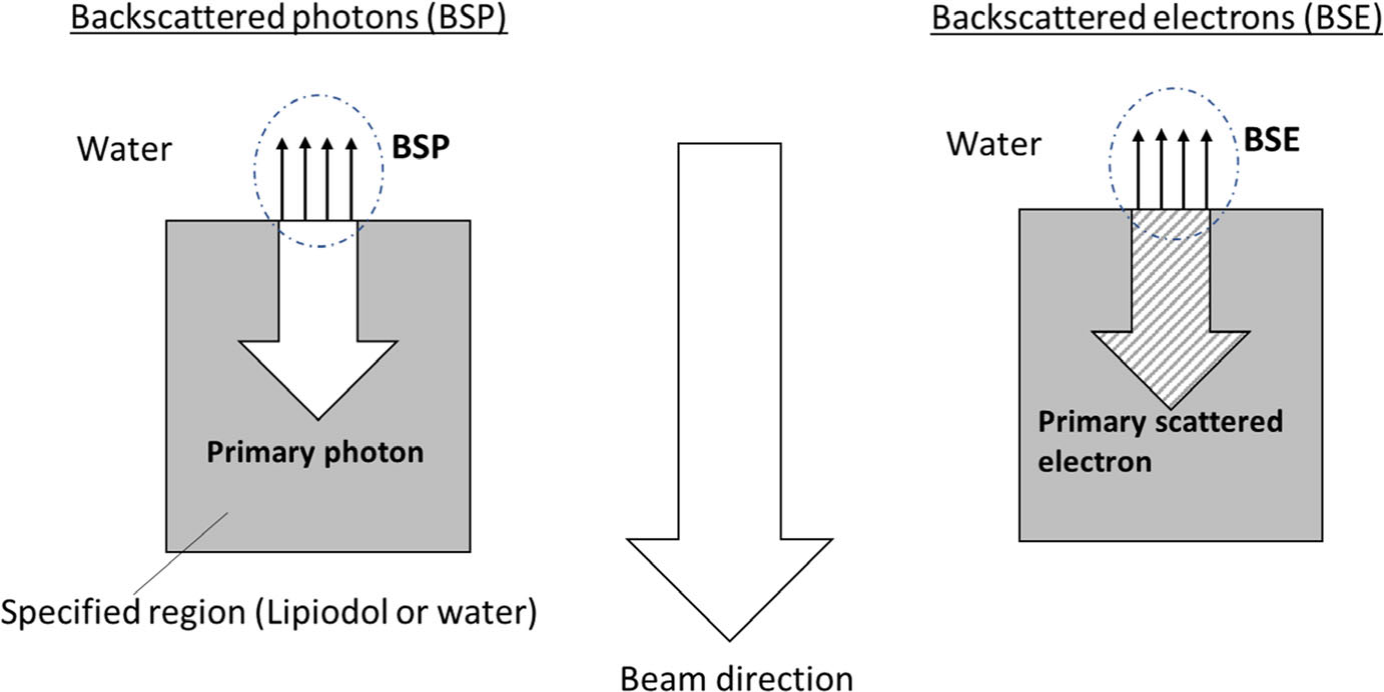
Definition of backscattered photons (BSP) and backscattered electrons (BSE).

The specified dose analysis aimed to calculate only the dose from the specified region, which was defined at 5–8 cm in this study. The specified dose analysis was performed by two methods, as shown in Fig. 2. One was the backscatter dose from the primary photons (BSP), and the other one was the backscatter from the primary scattered electrons (BSE). The ratio of the doses of BSP and BSE to total dose for each depth in steps of 0.05 cm in a range of 4.5–5.0 cm was analyzed.

## 3 RESULTS

### 3.1 Verification of MC calculation

**Figure 3:**
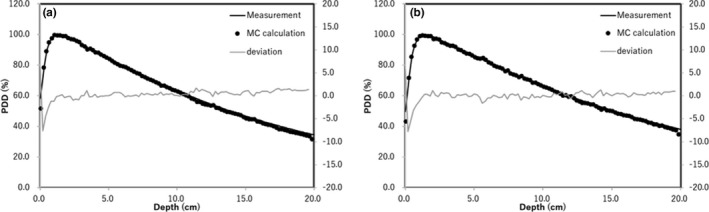
Validation of PDD curves comparing chamber measurement and MC calculation with (a) 6X FF beam and (b) 6X FFF beam.

Figure 3 shows the measured and MC‐calculated percent depth dose (PDD) for the 6X FF and FFF beams for a field size of 10 × 10 cm^2^. The MC‐calculated dose along the beam axis beyond the buildup point agrees with the measured dose within 1.0%.

### 3.2 Dose difference of dose and energy spectral variations of photons and electrons with virtual phantom

**Figure 4:**
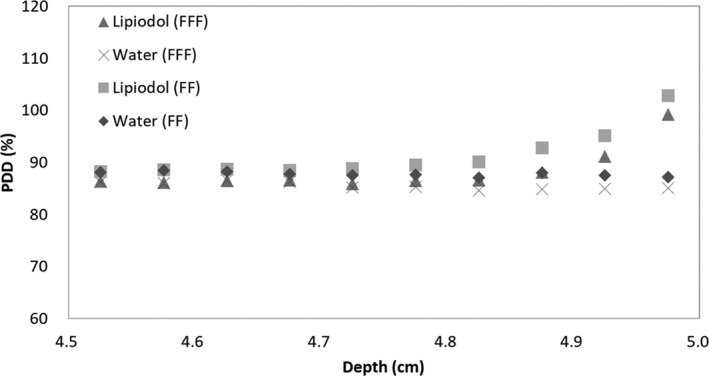
PDD with and without Lipiodol for 6X FFF and FF beams at a depth of 4.5–5.0 cm.

**Figure 5:**
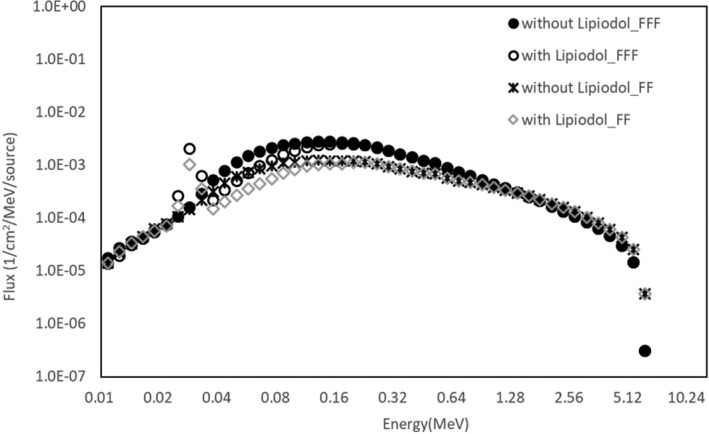
Photon energy spectra with and without Lipiodol in backscatter region (depth of 4.98 cm) for irradiation with 6X FFF and FF beams.

Figure 4 shows the PDD curves calculated with the MC method in the water phantom, with and without the Lipiodol, using 6X FF and FFF beams. All PDDs were normalized to 100% at d_max_ for each beam. In the dose calculation result, the dose difference with and without Lipiodol was 16.2% and 14.1% for the 6X FF and FFF beams at 4.98 cm, respectively. The dose difference decreased as the distance from the Lipiodol increased. The distance from the Lipiodol surface to the point where the deviation was observed with and without Lipiodol was within 3% (defined as the BD), and was 2.7 and 2.2 mm for the FF and FFF beams, respectively. The backscatter dose and the BD were larger for the 6X FF beam. Figure 5 shows the differences in the photon energy spectrum distributions with and without Lipiodol for 6X FFF and FF beams at a depth of 4.98 cm. The energy spectrum with Lipiodol contained more photons with energies mostly in the 0–0.3 MeV range, both for the 6X FF and the FFF beams. The FFF beam contained more photons with energies mostly in the 0–1.5 MeV range, while the FF beam contained more photons with energies mostly above 1.5 MeV.

### 3.3 Specified dose analysis in backscatter region

**Figure 6:**
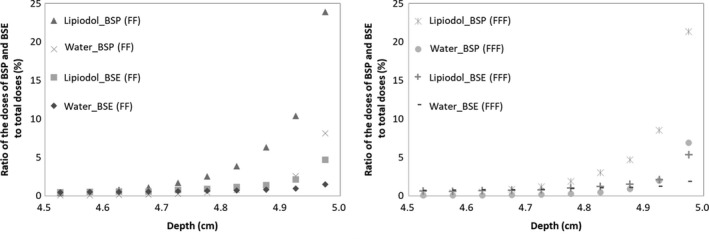
A ratio of the doses of BSP and BSE from specified region to the total dose with and without Lipiodol for 6X (left) FFF and (right) FF beams.

Figure 6 shows the ratio of the doses of BSP and BSE to total dose for each depth in steps of 0.05 cm in a range of 4.5–5.0 cm with and without Lipiodol for the 6X FFF and FF beams. The dose of BSP was larger than that of BSE both for the FFF and the FF beams at 4.7–5.0 cm of depth. The dose of BSP from the Lipiodol was larger than that from the water at a depth of 4.7–5.0 cm. The dose of BSP with the FFF beam was larger than that with the FF beam at a depth of 4.7–5.0 cm, while the difference in the BSE dose from Lipiodol and water was within 1% at all depths.

## DISCUSSION

The validation of MC calculation with water phantom with 6X FF and FFF beams was within 1%. Our past study showed the difference of the MC calculation and the measurement with 10X FFF beam was within 3%.^4^ The components of the Lipiodol was same as our previous study. Thus, the validation of the MC calculation with the Lipiodol could be used for the evaluation of the backscatter dose and energy spectrum from the Lipiodol. This study investigated the dose and energy spectrum of backscatter from Lipiodol, and the backscatter factor. The local dose enhancement was 16.2% and 14.1%, at maximum, for the 6X FF and FFF beams, respectively. As shown in Figs. 4 and 5, the local dose enhancement was affected by the dose of BSP, while the primary low‐energy photons at 0–0.3 MeV affected the backscatter. Therefore, the backscatter depends on the scatter of primary low‐energy photons. Berger et al. reported that the energy at the boundary between the photoelectric effect and the Compton scattering was higher for Lipiodol (0.3 MeV) than for water (0.03 MeV).^15^ Thus, a large number of lower photons was included by the photoelectric effect at 0–0.3 MeV. This was also demonstrated in our previous study, which analyzed the probability of electron generation in Lipiodol.^16^

The past study introduced metal fixed partial dentures, which caused the highest amount of dose enhancement (up to 33%).^17^ David reported that the range that water‐equivalent material almost completely absorbed the backscatter was 3–5 mm. In this study, the BD was within 3 mm, which is the same trend as that reported by David. Lipiodol has been used to treat liver cancer and remains partially or entirely in the tumor region.^18^ In the case where Lipiodol remains in the tumor region partially, the backscatter region can remain in the gross tumor volume (GTV). Moreover, if Lipiodol remains in the entire tumor region, the backscatter region is the outside of GTV. However, typically, a margin of at least 3–5 mm is added to the GTV to form the clinical target volume (CTV). This expansion was used in the RTOG 0438 Phase‐I trial for liver SBRT.^19^ The backscatter region is within 3 mm and therefore it would be included of the CTV. The past study demonstrates that commercially available gold nano particles cause radiosensitization and tumor growth delay with MV photons in tumor region.^20^ The current study used Lipiodol and it could cause the radiosensitization within CTV. With higher dose in the tumor region, dose escalation can be higher, while sufficiently sparing normal tissues surrounding the tumor. In other words, it would provide an option to achieve the prescribed tumor dose using a small amount of radiation for tumors managed well with current treatment planning protocol, thereby reducing the dose to surrounding normal tissues. Additionally, if the prescribed dose is not adjusted, the dose enhancement with Lipiodol could contribute to compensate the underdose in peripheral regions of the CTV for the dose‐blurring by patient setup error and organ motion.

A previous study investigated the difference in the surface dose with FFF and FF beams.^21^ The researchers reported that the FFF beam had a modestly higher surface dose in the buildup region in comparison with the corresponding FF beam for a field size ≤10 × 10 cm^2^. They concluded that the FFF beam contained lower‐energy photons in the buildup region, which increased the surface dose. In this study, we evaluated the backscatter dose from a high‐Z material, namely, Lipiodol. The backscatter dose was larger for the FF beam than for the FFF beam. The proportion of photons in the 0.04–0.64 MeV range was higher for the FF beam, which means that the energy spectrum of the FF beam was harder, as shown in Fig. 6. Almayahi reported that the backscattering increased as the photon energy increased.^22^ The difference between the photon and electron energy spectra between the FF and FFF beams was influenced by beam hardening caused by a flattening filter. The high‐energy photons contributed to the Compton scatter and increased the backscatter dose. From the result regarding the doses of BSP and BSE with Lipiodol, the difference in the backscatter dose between the FF and the FFF beams depended on the BSP difference. Therefore, in the backscatter difference of the FF and FFF beams, the high‐energy primary photons, rather than the low‐energy primary photons, contributed to the backscatter dose. Therefore, it follows that lower‐energy photons contributed to the backscatter difference between water and Lipiodol, while higher‐energy photons contributed to the cause the difference of the backscatters for FF and FFF beams. Thus, the factor of the backscatter depends on the variations between higher‐ and lower‐energy photons as a result of the material and primary photon energy. To clarify the factor of the backscatter, the energy and specified dose analyses should be performed as described in this paper.

Our study was limited with regard to calculating the Lipiodol density used in clinical applications, where the uptake patterns of Lipiodol around the tumor differ from case to case. In future work, we will focus on making the distribution of Lipiodol uptake similar to that in the CT value table. Moreover, we would like to estimate the backscatter dose by using the CT image of a clinical patient.

## 4 CONCLUSION

The backscatter effect was within 3 mm for both FFF and FF beams. The backscatter dose for FF beam was is larger than FFF beam. Additionally, the backscatter depended on the Compton scatter with primary photons. The backscatter dose can be used in clinical applications in cases where the backscatter region is within the tumor.

## CONFLICT OF INTEREST

The authors declare none.
